# Trust-Based Optimized Reporting for Detection and Prevention of Black Hole Attacks in Low-Power and Lossy Green IoT Networks

**DOI:** 10.3390/s24061775

**Published:** 2024-03-09

**Authors:** Muhammad Ali Khan, Rao Naveed Bin Rais, Osman Khalid, Sanan Ahmad

**Affiliations:** 1 Department of Computer Science, COMSATS University Islamabad, Abbottabad Campus, Abbottabad 22060, Pakistan; malikhan@cuiatd.edu.pk (M.A.K.); osman@cuiatd.edu.pk (O.K.); 2 Artificial Intelligence Research Center, Ajman University, Ajman 346, United Arab Emirates; 3 School of Science and Technology, University of Management and Technology, Lahore 54770, Pakistan; sanan.ahmad@netwrix.com

**Keywords:** green internet of things, black hole attack, RPL, low-power and lossy networks (LLNs), trust-based

## Abstract

The Internet of Things (IoT) is empowering various sectors and aspects of daily life. Green IoT systems typically involve Low-Power and Lossy Networks (LLNs) with resource-constrained nodes. Lightweight routing protocols, such as the Routing Protocol for Low-Power and Lossy Networks (RPL), are increasingly being applied for efficient communication in LLNs. However, RPL is susceptible to various attacks, such as the black hole attack, which compromises network security. The existing black hole attack detection methods in Green IoT rely on static thresholds and unreliable metrics to compute trust scores. This results in increasing false positive rates, especially in resource-constrained IoT environments. To overcome these limitations, we propose a delta-threshold-based trust model called the Optimized Reporting Module (ORM) to mitigate black hole attacks in Green IoT systems. The proposed scheme comprises both direct trust and indirect trust and utilizes a forgetting curve. Direct trust is derived from performance metrics, including honesty, dishonesty, energy, and unselfishness. Indirect trust requires the use of similarity. The forgetting curve provides a mechanism to consider the most significant and recent feedback from direct and indirect trust. To assess the efficacy of the proposed scheme, we compare it with the well-known trust-based attack detection scheme. Simulation results demonstrate that the proposed scheme has a higher detection rate and low false positive alarms compared to the existing scheme, confirming the applicability of the proposed scheme in green IoT systems.

## 1. Introduction

The Internet of Things (IoT) encompasses a diverse range of devices, including sensors, controllers, and actuators, that positively affect almost every aspect of life. The primary focus of traditional IoT is enabling connectivity and data exchange between devices to enhance efficiency, automation, and convenience in various applications. Although estimates for the number of connected devices vary, there is a consensus that the quantity and impact of these devices will be significant. Ericsson predicts that by the end of 2025, the number of connected devices will exceed 50 billion [1,2]. Recent years have experienced a paradigm shift from traditional IoT to Green IoT systems [3]. The key goal of Green IoT is to reduce energy consumption, minimize waste, and optimize the use of resources to have a lower impact on environment. However, implementing Green IoT solutions may be challenging due to limitations in resources and infrastructure. Security has become a major concern in Green IoT systems due to the lack of standardization and centralized control [4]. This deficiency in security could lead to a global security threat through continuous interaction and communication among compromised devices [5].

Green IoT systems are often categorized as Low Power and Lossy Networks (LLNs). These networks consist of devices with limited storage and battery resources, making communication among them highly susceptible to disruption [6]. Despite the implementation and improvement of various routing protocols, such as Routing Information Protocol (RIP), Open Shortest Path First (OSPF), and Interior Gateway Routing Protocol (IGRP), current protocols prove insufficient in tackling the crucial routing and security challenges encountered by LLNs. These challenges include packet loss, optimal path selection, and the need to concurrently address security vulnerabilities arising from compromised routing paths. Consequently, the existing protocols are not suitable routing options for most LLNs. To overcome this challenge, the Internet Engineering Task Force (IETF) recommends the Routing Protocol for Low-Power and Lossy Networks (RPL) routing protocol for LLNs, which efficiently integrates with resource-constrained devices [7]. Unlike other routing protocols, RPL searches for routes when needed rather than maintaining routing tables [6]. RPL is a proactive protocol based on a distance vector, utilizing a Destination-Oriented Directed Acyclic Graph (DODAG) topology, where all nodes connect to a single root node, known as a sink [8]. During DODAG construction, each node selects a preferred parent node based on performance metrics, ultimately serving as a gateway for leaf nodes. 

While RPL offers improved efficiency in terms of energy consumption and optimized data transmission, the security of LLNs is a major concern due to resource-constrained devices and the open nature of the protocol. Despite having Quality of Service (QoS) features, a secure operation mode is not enabled in RPL by default, making it vulnerable to various routing attacks [5], such as black hole, rank, wormhole, sybil, and hello flooding. These attacks have been discussed comprehensively in the literature [9]. Among these attacks, the black hole attack is one of the most harmful attacks in RPL, potentially leading to catastrophic consequences for any LLN network, such as unauthorized data access or illicit network manipulation. The black hole attack involves a malicious node falsely claiming the shortest path to all other network nodes, leading to the misdirection or dropping of transmitted data. Consequently, the impact of this attack may become more severe when all subsequent messages are barred from reaching the intended destination, resulting in an increased packet loss ratio and end-to-end delay in the network, thereby degrading the overall performance of the network. Furthermore, when multiple malicious nodes collaborate, the severity level increases significantly, a phenomenon known as a colluding black hole attack [6].

Several techniques have been proposed in the literature to detect and prevent black hole attacks, such as exponential smoothing-based [1], trust-based [8], encryption-based [10], and root-based [11] defense methods. Exponential smoothing-based methods are limited in providing comprehensive protection against evolving black hole attacks due to their inherent difficulty in adapting to rapidly changing attack patterns. Trust-based techniques struggle to create an efficient reporting module for isolating malicious nodes in Green IoT systems, primarily due to challenges in establishing and maintaining trust among resource-constrained devices. Encryption-based mechanisms are often deemed impractical for low resource environments, such as Green IoT, due to the resource-intensive and costly nature of encryption models. Finally, the root-based schemes, while effective in identifying malicious nodes, often rely on centralized decision making, which can be a bottleneck in large-scale LLNs and create single points of failure. Hence, there is a need to devise methods that improve network stability and efficiency. This paper addresses the black hole attack by proposing a mechanism to protect LLN integrity and enhance its performance. 

The proposed scheme outlines a comprehensive methodology for trust evaluation and propagation in an IoT network. It incorporates both direct and indirect trust mechanisms to assess node integrity, considering various parameters like energy, unselfishness, honesty, dishonesty, and similarity. The trust computation employs forgetting curves to prioritize recent behavior and mitigate the influence of old feedback on trust assessment. The trust propagation involves a delta (∆)-based strategy to determine the trustworthiness of nodes over time, allowing for the isolation of potentially malicious nodes. Compared to existing methods, this approach optimizes trust evaluation by avoiding immediate isolation, reducing root node feedback, and not limiting evaluations to specific timeframes. The proposed mechanism aims to counter the severity of black hole attacks, preventing packet loss, reducing end-to-end delay, and improving overall network performance. 

The key contributions of this work include the following:

1. The development of a novel trust-based Optimized Reporting Module (ORM) that alleviates the burden from the root node, preventing it from being a single point of failure. In the proposed scheme, the root node only receives the trust value of a node when action is required, rather than receiving trust values whenever they are calculated.
2. Reduction of the overhead cost associated with sending trust values of a specific node to the root within a fixed time span.
3. Implementation of RPL’s optimized routing algorithm to enhance network efficiency by mitigating latency, reducing overall transmission load, and lowering the burden on the root node.

The remainder of the paper is organized as follows. Section 2 discusses the related work. In Section 3, we discuss the classification of attacks on RPL-based IoT. The proposed scheme is presented in Section 4. Section 5 presents simulation results, comparisons, and performance evaluations. Finally, Section 6 concludes the paper.

## 2. Related Work

In recent years, numerous techniques have been proposed to mitigate malicious attacks on IoT networks. Some of the frequently used methodologies for mitigating black hole attacks in RPL-based IoT networks include acknowledgment-based methods, statistical/mathematical-based methods, specification-based techniques, and trust-based mechanisms [10].

Acknowledgment-based methods are utilized to detect and respond to black hole attacks through techniques like node marking or ICMPv6 echo messages. The authors in [12] present an acknowledgment-based security mechanism for combating black hole attacks in RPL networks within Cyber-Physical Systems (CPSs). It employs features like IPv6 over Low-Power Wireless Personal Area Networks (6LoWPANs) network discovery and avoids reliance on complex Intrusion Detection Systems (IDSs). Instead, a distributed timer-based approach is utilized for detecting malicious nodes.

Statistical/mathematical-based methods contribute to the detection and mitigation of black hole attacks by analyzing network traffic patterns, identifying anomalies indicative of suspicious behavior, and implementing algorithms to dynamically compute routing paths or isolate malicious nodes. In [13], the authors introduced an IDS focused on early detection and isolation of malicious nodes. This is achieved by leveraging local information shared among neighbors and employing a universal sink detection method in graph theory. The proposed system significantly enhanced the Packet Delivery Ratio and throughput, thereby preserving overall network performance.

Specification-based methods, as demonstrated by the security mechanism in Secure RPL, offer comprehensive approaches to identify and respond to black hole threats. In [14], the authors introduce GBG-RPL, a technique leveraging Gini-index and blockchain to detect and mitigate black hole attacks in smart health monitoring CPSs. GBG-RPL not only identifies anomalous activities but also proposes a resilient framework, achieving notable improvements in terms of reduced packet loss, enhanced residual energy utilization, decreased energy consumption, improved attack-detection rate, faster attack-detection time, optimized network management, and reduced end-to-end delay.

A trust-based mechanism involves the computation of trust values for each node by its neighboring nodes in the network. These trust values can be assessed and evaluated at various hierarchical levels. In the first case, all trust values can be propagated to the root, enabling it to take the necessary steps to maintain the integrity of the network [15]. In another scenario, the entire network can be divided into equal clusters [16]. The trust value of each node in a cluster is then transmitted to the cluster’s head (CH). Subsequently, the CH assumes the responsibility of directing these trust values to the Base Station (BS) of the network. In the last case, only the parents in the entire network topology are monitored, and their trust values are then propagated to the root.

Several trust-based models have been proposed in the literature to identify and isolate compromised nodes within the network, ensuring the preservation of the network’s integrity. To isolate malicious nodes, a trust score is computed for each node, which is subsequently compared either within a fixed time span or without any time constraints against a pre-defined threshold. In addition to trust-based models, several alternative schemes have also been proposed. However, these schemes lack an optimized method for efficiently propagating the credibility of a specific node to the network’s root.

The authors in [17] propose a blockchain-based trust model for the Wireless Sensor Internet of Things. Using the Dijkstra algorithm, an efficient routing protocol is introduced to prevent void holes between ordinary sensor nodes and a sink node. All transactions are recorded in an immutable blockchain for transparency, employing the Proof of Authority consensus algorithm, which outperforms the proof-of-work algorithm. In a study by Ahmad et al. [18], a mitigation procedure was suggested for isolating black hole nodes in the network. In this technique, all neighboring nodes report the suspicious node to the root of the network. Subsequently, it becomes the responsibility of the root node to scrutinize the routines or activities of the suspicious node. If the suspicious node is confirmed as malicious, the root instructs all neighboring nodes to cease communication with that particular node. A drawback of this technique is the extended time required before the compromised node is effectively isolated.

Sahay et al. [1] proposed an exponential smoothing-based approach, wherein the activities of all nodes are recorded. The objective of recording these activities is to identify the last time a specific node transmitted a packet to the root. In the end, the root examines whether any node surpasses the predefined time. If so, it is concluded that the node is malicious. However, a weakness of this approach lies in the potential for a significant number of false positives. For instance, a node experiencing communication or other issues may be incorrectly identified as malicious.

In [10], the authors presented a technique named Encrypted Data Packets, which utilized different encryption mechanisms. A significant drawback of this method is that it increases the packet response time, as all packets are encrypted in this process. Gautham et al. proposed a scheme named the Robust Trust Model (RTM) [19], which enhances the security of RPL by identifying the most secure path in the network. However, a weakness of this technique is its high energy consumption, leading to a degradation in network performance. Furthermore, the reporting mechanism employed in RTM is not suitable, resulting in a higher occurrence of false positives. Airehrour et al. proposed a technique for selecting the best parent node using the trust-based threshold mechanism against rank modification attacks [8]. The advantage of this technique is the early isolation of any malicious node during parent node selection, effectively mitigating rank attacks. However, a limitation of this method is its ineffectiveness in addressing other routing attacks, such as the black hole attack.

Sun et al. proposed a theoretical methodology for quantitatively measuring trust based on two axioms [20]. The first is referred to as the entropy-based model, while the second is named the probability-based model. Both models fulfill their respective axioms. The advantage of this technique is that these two models can enhance the network’s throughput. However, a challenge associated with these models is the extensive computation required to satisfy the axioms. Zhang et al. [21] classified the determination of trust metrics in a network into being elicited through a direct trust or an indirect trust technique. A trust-based model called Logistic Trust is proposed in [22]. It is established based on the source node’s communication experience and the recommendations provided by the neighboring nodes of the target node. Logistic Trust employs a function known as the Logistic Function (LF), which integrates both direct and indirect trust to identify malicious nodes in the network. The main issue with this technique is that both the calculated direct and indirect trusts are compared to a predefined threshold value. If any node’s packet loss ratio exceeds this threshold, then that node is considered malicious. However, there could be various reasons for a poor packet loss ratio that are not indicative of the node being compromised. 

In [23], the authors proposed a dynamic trust model based on a time decay factor. The method calculates the trust value of a node considering various factors, such as interaction, attribute similarity, public friend count (the number of mutual friends), and the time factor, with particular emphasis on the time factor, which significantly influences the trust value of the nodes. When a node lacks interaction with other nodes in the network, its trust value decreases. The proposed model computes the trust value of each node using the direct trust computation method. Similar to the methodology in [22], a node may be identified as a malicious node if it fails to participate in a communication process within a specific time due to network issues. When a node is not communicating with other nodes, its number of interactions decreases. Consequently, the public friend count factor is also likely to decrease for that node.

Marchang et al. proposed a lightweight trust-based routing protocol to mitigate black hole attacks in mobile ad hoc networks (MANET) [24]. Instead of opting for the shortest route as in traditional routing, preference is given to the most trusted path. Each node maintains the trust value for its neighbor nodes, indicating the level of trust they have in these neighboring nodes. In this model, the trust value for a path is calculated based on the activities completed by interlinked neighbor nodes. However, a drawback of this model is the excessive consumption of network energy, as each node in the network computes the trust value for its neighbor nodes.

Lahbib et al. [25] focused on integrating a trust model into the RPL routing protocol. During the construction of the network topology, several crucial steps were implemented to secure the network from both external and internal attacks. The model emphasizes the computation of both link and node trust through a multi-dimensional approach. The evaluation of trust involves four stages: information gathering; trust composition (integrating both node and link trust); trust database creation; and trust application. Trust calculation is matrix-based, with influencing factors including the level of energy, positive interaction, and node satisfaction. When compared to existing approaches, their model demonstrates greater accuracy in detecting and isolating malicious nodes. However, challenges persist regarding network delays.

As discussed above, the existing schemes employ various methods for reporting trust scores in the identification and isolation of malicious nodes within the network. In some of the schemes, if a specific node’s trust score is reported and found to be below a predefined threshold, immediate isolation of the node follows. Conversely, in other approaches, if the node’s trust score remains below the predefined threshold for a specific duration, the node is then isolated. The issue with the former approaches is that the trust value of a node can be adversely affected by having malicious neighbor nodes or encountering different network issues, rendering it an unreliable approach for isolation. The latter methodologies also present multiple challenges. There is a possibility that the node’s trust value may not be sufficient within a short time span. Moreover, extending the time span for the root to gather more feedback results in an increase in computation cost and overhead. Such escalation in computation cost and overhead renders the mechanism unsuitable for resource-constrained nodes in an IoT-based network. Furthermore, with greater numbers of feedback, more memory is consumed at the root or CH. Therefore, a method needs to be devised to reduce the computation cost for nodes in the network and minimize overhead. It is crucial not to isolate a node from the network solely based on the immediate feedback received by the root if its trust score is below the threshold. This is because frequent variations in topology can also compromise the stability of the network. Table 1 compares the existing schemes for various parameters.

## 3. Classification of Attacks on RPL-Based IoT

Attacks targeting RPL-based IoT networks can be categorized into two primary classes: (a) the type of attacks stemming from vulnerabilities inherent in RPL itself and (b) those inherited from Wireless Sensor Networks (WSNs) [10]. Some of the common RPL-specific attacks encompass rank attacks, version attacks, DODAG Information Solicitation (DIS) attacks, neighbor attacks, and replay attacks. Attacks inherited from traditional WSNs include black hole and selective forwarding attacks, sinkhole attacks, wormhole attacks, Sybil attacks, and hello flood attacks. While these attacks have their roots in WSNs, their methodologies have evolved to adapt to the unique challenges posed by the IoT paradigm. These attacks are briefly discussed in the following subsections.

### 3.1. Wireless Sensor Network-Inherited Attacks

#### 3.1.1. Black Hole Attacks

**Figure 1:**
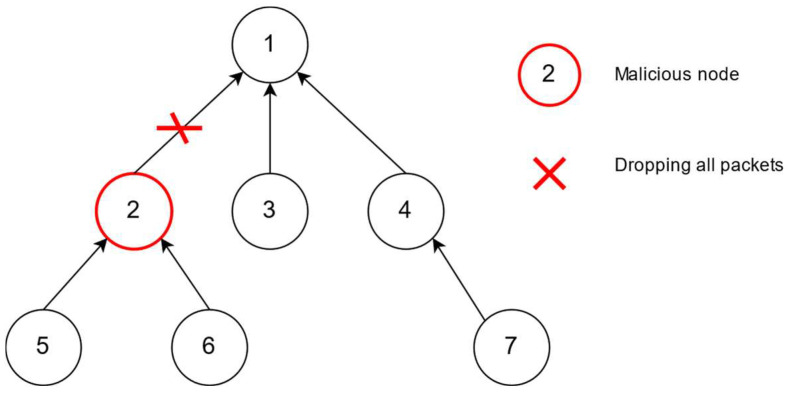
RPL-based IoT network with black hole attack. Malicious node 2 drops all received packets from nodes 5 and 6.

A black hole attack, a type of Denial of Service (DoS) attack, occurs when a malicious node intentionally discards all received packets instead of forwarding them as shown in Figure 1. An advanced variation, known as a Selective-Forwarding attack, involves malicious nodes selectively forwarding RPL control messages while discarding other packets. These malicious nodes typically participate in the creation and maintenance of DODAG, mimicking the behavior of benign nodes.

The malicious node propagates false information in the network, thereby misleading nearby nodes about its capabilities. This includes false advertisements claiming to have the shortest path to the destination, thus convincing the neighboring nodes to route their packets through the malicious node [38]. Upon joining the routing path, the malicious node intentionally drops all received packets, disrupting communication among legitimate nodes. 

The consequences of a black hole attack are significant, leading to disruption either in a specific network segment or across the entire network, especially with the presence of multiple malicious nodes. Conversely, Selective Forwarding is a less aggressive form of attack, occasionally disrupting routing paths. The propagation mechanism of a black hole attack causes the legitimate nodes to unknowingly route their packets through the malicious node. This disruption adversely impacts network performance and can lead to an overall degradation of system functionality [12].

#### 3.1.2. Sinkhole Attacks

In sinkhole attacks, malicious nodes attract network traffic by broadcasting deceptive routes with superior metrics, causing nearby nodes to route their traffic through these adversaries [39]. This attack can be executed by transmitting a DODAG Information Object (DIO) control message with enhanced rank and Objective Function (OF) parameters, by manipulating preferences, or by redirecting passing traffic toward another adversary, thus creating a sinkhole.

#### 3.1.3. Wormhole Attacks

In a wormhole attack, two malicious nodes collaborate to create a tunnel between them, diverting network traffic through this tunnel instead of the regular DODAG path. This can be achieved through packet encapsulation, packet relay, or an out-of-band link. The attack allows adversaries to deceive the network, manipulate hop counts, and establish covert communication channels [40].

#### 3.1.4. Sybil Attacks

In a Sybil attack, malicious nodes adopt the identity of legitimate nodes, often using multiple identities per malicious node. Sybil attacks are classified into SA-1, focusing on data manipulation in a fixed area; SA-2, distributed among nodes to disrupt routing and manipulate reputations; and SA-3, similar to SA-2 but with mobile nodes, making detection more challenging [41]. Malicious nodes can use stolen or fabricated identities for communication, with the option of simultaneous or non-simultaneous deployment of multiple identities. 

#### 3.1.5. Hello Flood Attacks

In a Hello Flood attack, a malicious node exploits the network entry process, typically initiated with a “HELLO” message or DIO message in the case of RPL. The attacker sends deceptive DIO messages with robust routing metrics or a strong signal, then abruptly disappears or lowers its transmission power. It can interrupt the delivery of packets from legitimate nodes, causing depletion of their resources and escalating network congestion due to increased control overhead [38].

### 3.2. RPL-Specific Attacks

#### 3.2.1. Rank Attacks

Rank attacks involve adversaries manipulating the Rank and Objective Function (ROF) values distributed by DIO messages. Within the category of rank attacks, adversaries may employ decreased rank attacks, advertising lower ranks to attract nodes as preferred parents, or increased rank attacks, advertising higher ranks near the root node to disrupt routing topology [42].

#### 3.2.2. Version Attacks

This attack exploits RPL’s Global Repair feature, manipulating the version number of the DODAG to induce topology inconsistency and routing loops. In the absence of mechanisms ensuring version number integrity, a malicious node can initiate the global repair procedure by transmitting a DIO message with a higher version number [43]. The primary objective of version number attacks is to deplete node resources and impede packet delivery.

#### 3.2.3. Neighbor Attacks

In this attack, an adversary forwards received DIO messages to neighboring nodes without modification, creating a false impression of the original sender being within the range of those nodes. While the impact of this attack alone results in a slight increase in end-to-end delay, its significance grows when combined with other attacks [44]. 

#### 3.2.4. Replay Attacks

These attacks involve capturing and forwarding legitimate control messages (DIO, DIS, and Destination Advertisement Object (DAO)) within the network. They focus on control messages, setting them apart from similar attacks in WSNs. Replay attacks have a more pronounced impact in dynamic networks, where adversaries can record and replay control messages to force neighboring nodes into updating routing tables with outdated information [45].

## 4. Proposed Trust-Based Optimized Reporting Scheme

This section explains the proposed trust-based optimized reporting scheme, with the fundamental concepts illustrated in Section 4.1, focusing on key elements such as Trustor, Trustee, and Recommenders and their relationships with the Group Member Nodes (GMNs) and Group Leader Nodes (GLNs). The trust calculation mechanism is detailed in Section 4.2; it utilizes both direct and indirect trust assessments and considers factors such as energy, unselfishness, honesty, dishonesty, and similarity. Finally, in Section 4.3, we discuss the Delta-Based Isolation Strategy, revealing how it determines whether the trust value of a specific trustee is decreasing and its implications for optimizing cost and reducing root node overhead in trust management.

### 4.1. Fundamental Concepts in Trust Management

- Network Structure: We consider an IoT network where all nodes can communicate and exchange information with each other. The network primarily consists of two types of nodes: GMN and GLN. GMNs are entities within the network seeking to establish trust, while GLNs play a crucial role in trust management. GLNs collect the computed trust scores for each GMN and decide whether specific GMNs are trustworthy to be a part of the network. In the proposed model, we represent each communication request as a “task” in the subsequent text.
- Trustor: This refers to entities within the network that seek to evaluate and establish trust in other network participants, including GMNs and GLNs. In our context, Trustors are nodes or devices within the IoT network that engage in the process of trust calculation for both GMNs and GLNs.
- Trustee: This represents the entities being evaluated for trustworthiness, including both GMNs and GLNs. Trustees may be individual nodes, devices, or even entire subsystems in the IoT network. Understanding the characteristics and behaviors of Trustees is crucial for effective trust evaluation and management.
- Recommenders: These act as intermediaries that contribute to the trust evaluation process for both GMNs and GLNs. These entities provide valuable insights and recommendations based on their observations of Trustors and Trustees, thereby enhancing the robustness of trust calculations with the network.

### 4.2. Trust Calculation Mechanism

The proposed methodology utilizes both direct and indirect trust-based assessments. The integrity of each node in the system is assessed by considering various factors, including energy, unselfishness, honesty, and dishonesty, and utilizing a forgetting curve for direct trust evaluation. In addition to these factors, similarity is also employed for indirect trust calculation to preserve the integrity of the network.

#### 4.2.1. Notations and Their Meanings

- Energy ($T_{e})$: This represents the residual energy of a specific node to participate in communication in the network. It is calculated by subtracting the current energy from the initial energy.
- Unselfishness ($T_{u})$: This indicates how much a node has responded to transmission requests from neighboring nodes. It involves the trust value for energy and the number of successful communications.
- Honesty ($T_{h})$: This assesses the node’s trustworthiness based on the number of completed transmissions.
- Dishonesty ($T_{dh})$: This assesses the node’s trust based on the number of unsuccessful transmissions.
- Forgetting curve: This is a mechanism that prioritizes recent behavior over older feedback in trust evaluation.
- Similarity ($T_{s})$: This measures the variation among the feedback propagated by the recommenders.
- $E_{i}$: a node’s initial energy.
- $E_{c}$: a node’s current energy.
- ${NT}_{d}$: the direct trust of a node.
- ${NT}_{i}$: the indirect trust of a node.
- ST: the number of successful tasks. In this paper, we represent each communication request as a “task”.
- UT: the number of unsuccessful tasks.
- $F_{h}$: a set of honest feedback.
- $F_{dh}$: a set of dishonest feedback.
- $T_{c}$: the current trust value.
- $T_{i}$: the initial trust value.

#### 4.2.2. Direct Trust

When a source node (trustor) interacts with its immediate neighbor nodes (trustees) in the network, the direct trust can be calculated using [26] through a consideration of energy-related parameters. The direct trust ${NT}_{d}$ is calculated by the combination of energy, unselfishness, honesty, and dishonesty trust values, as shown in (1):

(1) $${NT}_{d}=T_{e}+T_{u}+T_{h}-T_{dh},$$

where ${NT}_{d}$ refers to the direct trust, $T_{e}$ is the energy trust value, $T_{u}$ is the unselfishness trust value, $T_{h}$ is the honesty trust value, and $T_{dh}$ is the dishonesty trust value. The trust value $T_{e}$ refers to the residual energy of the specific node in the network. It can be calculated by subtracting the current energy $E_{c}$ of the node from its initial energy $E_{i}$, as in (2).

(2) $$T_{e}=E_{i}+E_{c}.$$

The trust value of unselfishness $T_{u}$ can be computed using the trust value for energy and the number of successful tasks (STs) out of a total “n” communication, as shown in (3).

(3) $$T_{u}=T_{e}\times ST.$$

**Figure 2:**
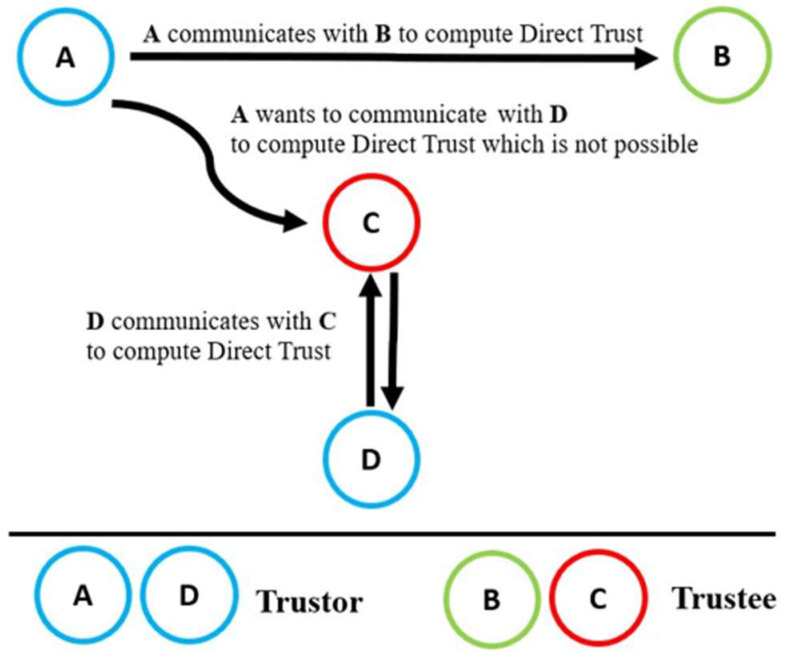
Direct trust calculation: node A assesses node B’s trustworthiness.

Both of the energy-aware parameters, i.e., energy ($T_{e}$) and unselfishness ($T_{u}$), are significant in evaluating the trustworthiness of nodes in communication in the network. This approach is designed to minimize energy consumption during trust evaluations, contributing to overall cost efficiency. As illustrated in Figure 2, node A would exhibit a lower trust value for unselfishness toward node B if node B fails to respond to pending tasks despite having sufficient energy. This energy-centric approach ensures that the trustworthiness assessment incorporates considerations for the nodes’ energy reserves and responsiveness, promoting an energy-efficient trust calculation mechanism within the IoT network.

Conversely, if node B responds to pending tasks from node A with nominal energy, node A would assign a higher trust value for unselfishness to node B. Additionally, node A calculates the trust values for honesty ($T_{h}$) and dishonesty ($T_{dh}$) for node B based on the number of successful tasks (STs) or unsuccessful tasks (UTs) completed by node B. Equations (4) and (5) describe the honesty and dishonesty parameters more precisely.

(4) $$T_{h}=\frac{ST}{ST+UT}.$$

(5) $$T_{dh}=\frac{UT}{ST+UT}.$$

Trust value for honesty ($T_{h}$) is computed by dividing the number of successful tasks by the total number of tasks. Similarly, the trust value for dishonesty ($T_{dh}$) is evaluated by dividing the number of unsuccessful tasks by the total number of tasks. The direct trust (${NT}_{d}$) of each node would be higher if the trust value for $T_{h}$ and the trust value for $T_{dh}$ is low, and vice versa. 

Algorithm 1 computes the direct trust (${NT}_{d}$) for a target node based on its trustworthiness regarding energy, unselfishness, honesty, and dishonesty. It checks if the target node is an immediate neighbor and verifies the existence of trust values associated with it. If absent, ${NT}_{d}$ is calculated using formulas involving energy ($T_{e}$), unselfishness ($T_{u}$), honesty ($T_{h}$), and dishonesty ($T_{dh}$). The algorithm keeps track of the cumulative direct trust values (${NT}_{d}$) for each node ID and counts the computations conducted for individual nodes. Once ${NT}_{d}$ is calculated, it updates the direct trust values using a mechanism that prioritizes recent assessments over older ones. Ultimately, this algorithm assesses the direct trustworthiness of a target node within the network based on specific trust parameters, employing a system that values recent evaluations. By evaluating energy and unselfishness, the direct trust calculation is optimized to minimize costs associated with excessive energy consumption during trust assessments.
**Algorithm 1:** Computing Direct Trust**input:** Trust values of $T_{e}$, $T_{u}$, $T_{h}$, $T_{dh}$
**output:** Direct Trust ${NT}_{d}$
      **if** Target node is an immediate neighbor node **then**          **if** Trust value does not exist for Target node **then**                 ${NT}_{d}=T_{e}+T_{u}+T_{h}-T_{dh}\;NTD\left[{node}_{id}\right]=NTD\left[{node}_{id}\right]+{NT}_{d}$          **else**                 
${NT}_{d}=T_{e}+T_{u}+T_{h}-T_{dh}$                 $NTD\left[{node}_{id}\right]=NTD\left[{node}_{id}\right]+{NT}_{d}\;Counter\left[{node}_{id}\right]=Counter\left[{node}_{id}\right]+1\;forgettingCurve(NTD,Counter,{node}_{id})$          **end if**
     **end if**

#### 4.2.3. Indirect Trust

When a source node (trustor) initiates a communication with the target node (trustee), which is not its immediate neighbor, then the concept of indirect trust is utilized [27]. Node A computes indirect trust for node E using the following equation:

(6) $${NT}_{i}=T_{e}+T_{u}\pm T_{s}.$$

Here, ${NT}_{i}$ refers to the indirect trust calculated by the combination of energy, unselfishness, and similarity ($T_{s}$) trust values, as shown in (6). Similarity ($T_{s})$ refers to the measure of variation among the feedback propagated by the recommenders. This parameter helps the trustor to assess whether the opinions about a specific trustee are centralized or dispersed. If the feedback is more centralized, it becomes easier for the trustor to form an opinion (honest/dishonest) about the targeted node, and vice versa. The similarity trust value is computed using assessments of reliability and trustworthiness based on received feedback. The similarity of feedback for any specific node can be calculated using the following equation:

(7) $$T_{s}=\frac{max({|F}_{h}|,{|F}_{dh}|)}{{|F}_{h}|+{|F}_{dh}|}.$$

The numerator in (7) determines the higher count between honest feedback ($F_{h}$) and dishonest feedback ($F_{dh}$). Whereas the denominator represents the combined count of both honest and dishonest feedback, encompassing all received feedback values. If there is a greater number of honest feedback values regarding the target node, $T_{s}$ is added in (6). Conversely, $T_{s}$ is subtracted in (6) when the number of dishonest feedback values outweighs the honest feedback. There is a possibility that the source node receives an equal number of honest and dishonest feedback values. In such a case, $T_{s}$ is assumed to be 0 because the amount of honest and dishonest feedback is equal. Consequently, the indirect node trust relies solely on energy and unselfishness when the similarity factor nullifies itself due to an equal amount of honest and dishonest feedback. 

Algorithm 2 focuses on evaluating the indirect trust of a target node within the network. It begins by checking whether the target node is an immediate neighbor or not. If the node is not an immediate neighbor, it proceeds to compute the indirect trust. If the trust value does not exist for the target node, the algorithm calculates the indirect trust based on the values related to energy, unselfishness, and similarity. This computed value is then updated in the system records. Conversely, if a trust value already exists for the target node, the algorithm recalculates the indirect trust using the same parameters and updates the relevant records accordingly. Additionally, it employs a counter mechanism and a forgetting curve function to adjust the stored indirect trust values, contributing to the ongoing trust assessment process in the network.
**Algorithm 2:** Computing Indirect Trust**input:** Trust values of $T_{e}$, $T_{u}$, $T_{s}$ **output:** Indirect Trust ${NT}_{i}$      **if** Target node is an immediate neighbor node **then**           **if** Trust value does not exist for Target node **then**                   ${NT}_{i}=T_{e}+T_{u}\pm T_{s}\;NTI\left[{node}_{id}\right]=NTI\left[{node}_{id}\right]+{NT}_{i}\;Counter\left[{node}_{id}\right]=Counter\left[{node}_{id}\right]+1$
        **else**                   ${NT}_{i}=T_{e}+T_{u}\pm T_{s}\;NTI\left[{node}_{id}\right]=NTI\left[{node}_{id}\right]+{NT}_{i}\;Counter\left[{node}_{id}\right]=Counter\left[{node}_{id}\right]+1\;forgettingCurve(NTI,Counter,{node}_{id})$
         **end if**
     **end if**

#### 4.2.4. Forgetting Curve

**Figure 3:**
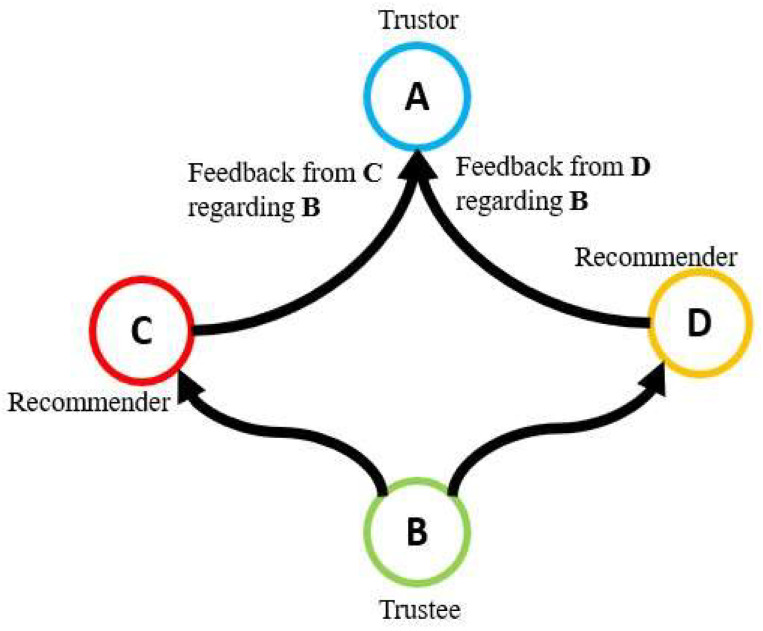
Indirect trust computation: nodes C and D contribute feedback about node B to node A.

Unlike direct trust, the computation of indirect trust relies on the feedback provided by the recommenders to the source node. As illustrated in Figure 3, nodes C and D provide their feedback regarding node B to the trustor node A. Node A receives separate feedback based on $T_{e}$ and $T_{u}$. The trust value for $T_{s}$ is evaluated at node A once it receives all the feedback. 

A forgetting curve/time decay function is employed based on [28], prioritizing the consideration of recent trust scores for the specific node over old feedback received by the trustor. The forgetting function is crucial for authentic trust evaluation, as it gives greater prominence to the recent behavior of the targeted node.

As depicted in Figure 3, node A receives numerous feedback values from nodes C and D concerning node B. Node A assesses the credibility of trustee node B using a fixed number of feedback values. When the number of feedback values equals the predefined threshold, the calculated trust values are filtered out. Node A continues to propagate the previously computed total trust for node B to the GLN. The operation of the forgetting function for both direct and indirect trust calculation is outlined in Algorithm 3.
**Algorithm 3:** Forgetting Curve**input:** t—Direct trust or Indirect trust values, c—Counter, n—${node}_{id}$ **output:** Setting the priority of recent test scores **function**
$forgettingCurve\left(t,c,n\right):$
      **if**
$c\left[n\right]==threshold$
**then**           $t_{root}(t\left[n\right])$          $t\left[n\right]=0$          $c\left[n\right]=0$   **end if**   **if**
$c\left[n\right]<threshold$
**then**          $t_{root}(t\left[n\right])$   **end if** **return**

Algorithm 3 involves prioritizing recent trust scores in the system. It considers inputs like direct or indirect trust values, counters, and specific node identifiers. If the counter for a node reaches a defined threshold, it invokes a function to update the trust value at the root node. This process resets the trust and counter values for that node. Alternatively, if the counter is below the threshold, it still triggers the function to update the root node with the current trust value for the node under consideration. Finally, the algorithm ensures that trust updates are sent to the root node based on defined thresholds for ongoing network trust assessment.

### 4.3. Trust Propagation

As discussed earlier, the purpose of trust computation is to determine whether a specific node is trustworthy enough to be included as part of the network. To take preventive action against malicious nodes, a propagation mechanism is also necessary to circulate the updated trust value toward the root or CH of the network for the corresponding action. In some existing works, if the trust score of a specific node is reported to be less than a predefined threshold once, at most, the node is isolated [29]. In others, if the trust score of the node remains below the predefined threshold for a specific period, then the node is isolated [30]. Therefore, this work proposes a delta (∆)-based optimized methodology for isolating and reporting the trust value of a malicious node. Each source node (trustor) maintains the value of delta (∆) for each target node (trustee) with which it communicates. The following expressions depict the value of delta.

(8) $$\Delta_{o}=T_{c}-T_{i}.$$

(9) $$\Delta_{1}=T_{c}-\Delta_{o}.$$

(10) $$\Delta_{n}=T_{c}-\Delta_{n-1}.$$

where $T_{i}$ represents the initial trust value and $T_{c}$ indicates the current trust value.

#### 4.3.1. Delta-Based Isolation Strategy

In this subsection, the computation of delta values is introduced as a mechanism to investigate whether the trust value of a specific trustee is decreasing. The delta values are calculated based on the difference between the current trust value ($T_{c}$) and the initial trust value ($T_{i}$). In (8), the parameter $\Delta_{o}$ represents the initial value of delta, which a source node (trustor) computes for any specific target node (trustee). In (9), $\Delta_{1}$ denotes the secondary value of the delta, calculated using the primary delta ($\Delta_{o}$). Finally, the expression (10) illustrates this continuous process until the node is isolated. The purpose of computing this delta is to investigate whether the trust value of a specific trustee is decreasing. The value of delta depends solely on the current trust of the target node. If the current trust is less than the initial trust in (8) or the primary trust in (9), then the delta value is considered low, and vice versa. Once the delta value of a specific target node continuously decreases a fixed number of times (a predefined threshold), the node is reported to the root for isolation. Consequently, the node is isolated from the network, and its delta values are flushed out from all concerned source nodes in the network.

#### 4.3.2. Cost Optimization through Delta-Based Strategy

This subsection elaborates on how the delta-based isolation strategy presents a cost-effective approach to trust management. Unlike immediate isolation upon falling below a threshold, the methodology minimizes unnecessary isolation events by focusing on the trust degradation trend over time. This reduces the potential for false positives, ensuring that nodes are isolated based on sustained untrustworthiness rather than momentary fluctuations. By adopting this strategy, the proposed methodology optimizes costs associated with unnecessary node isolation, enhancing the overall efficiency of trust management in IoT networks.

#### 4.3.3. Root Node Overhead Reduction

Here, we highlight how the proposed methodology optimizes cost by minimizing the burden on the root node. As outlined in Algorithm 3, the forgetting curve mechanism, coupled with a predefined threshold, filters out calculated trust values after a certain number of feedback values are received. This strategic design choice ensures that the root node is spared from processing excessive feedback, leading to more efficient resource utilization. The reduced load on the root node is particularly valuable for large-scale IoT networks, where minimizing overhead is crucial for scalability. Thus, the proposed methodology not only enhances trust management but also strategically reduces the overall costs associated with root node processing and memory consumption.

## 5. Simulation and Results

To demonstrate the efficiency of the delta-based trust model, we compare our proposed methodology with the Tree-based Trust Dissemination (TTD) method [31]. This involves a trust-based IDS for IoT networks, centered on establishing trust relations among nodes in a DODAG. Nodes compute trust values based on observed behavior, employing belief, disbelief, and uncertainty variables to assess neighbors’ actions. The trust evaluator monitors message forwarding, rank consistency, and version number updates to adjust trust values accordingly. These values are shared and aggregated by a border router or CH using the Subjective Logic consensus operator. Three algorithms are proposed for reputation management, allowing centralized or distributed trust calculation within the network. Overall, the approach aims to detect potential intruders by evaluating and combining trust values, facilitating proactive responses to malicious activities while ensuring secure message routing in the IoT network.

Additionally, we compared our proposed methodology with the technique “Mitigation of black hole attacks in 6LoWPAN RPL-based Wireless sensor network (RWC)” as proposed in [39]. This method utilizes a two-step verification process to detect and mitigate black hole nodes within RPL-based WSNs. Initially, nodes scrutinize their parent nodes for any suspicious activities, flagging them as potential black holes if they fail to receive data packets within a predefined time interval. Subsequently, neighboring nodes cooperate to validate the integrity of these suspected nodes, taking corrective actions if the verification process indicates a failure. This methodology effectively reduces network overhead and enhances the reliability of black hole detection, even under challenging network conditions.

### 5.1. Performance Metrics

The experiments are performed to evaluate the efficiency of the proposed system in differentiating between benign and malicious nodes. The following metrics provide a quantitative measure of the system’s accuracy, sensitivity, specificity, and overall discriminatory power. Each metric plays a crucial role in comprehensively evaluating the system’s performance and is fundamental to the subsequent analysis.

- True Positive (TP): the number of intruder nodes correctly recognized as malicious by the detection system.
- True Negative (TN): the number of benign nodes correctly identified as non-intruders by the detection system.
- False Negative (FN): the number of malicious nodes mistakenly categorized as benign by the detection system.
- False Positive (FP): the number of benign nodes mistakenly classified as malicious by the detection system.
- True Positive Rate (TPR): also known as sensitivity, this represents the proportion of actual positive instances (intruder nodes) that are correctly identified by the system.

(11) $$TPR=\frac{TP}{TP+FP}.$$

- False Positive Rate (FPR): the proportion of actual negative instances (benign nodes) that are incorrectly identified as positive (misclassified as intruders).

(12) $$FPR=\frac{FP}{TN+FP}.$$

FPR can also be represented as

(13) FPR=1−Specificity,

where specificity is known as true negative rate, defined as follows.

- True Negative Rate (TNR): also known as specificity, this signifies the proportion of actual negative instances (benign nodes) that are correctly identified by the system.

(14) $$TNR=\frac{TN}{TN+FP}.$$

- Receiver Operator Characteristic (ROC) Curve: The ROC curve is a graphical representation that illustrates the trade-off between TPR and FPR at various thresholds. It helps evaluate the performance of a classification model across different operating points.

### 5.2. Results

We implement a black hole attack in the RPL protocol, which is a critical threat to IoT networks, by utilizing the Contiki operating system within the Cooja simulator. This investigation occurs within the 6LoWPAN IoT environment, focusing on the execution and analysis of black hole attacks to understand their implications thoroughly. Moreover, our study introduces and assesses dedicated detection and prevention algorithms tailored to counter black hole attacks within RPL-based IoT systems. This research serves to demonstrate the effectiveness of our approach in strengthening the detection and mitigation of such malicious activities in the realm of IoT security.

**Figure 4:**
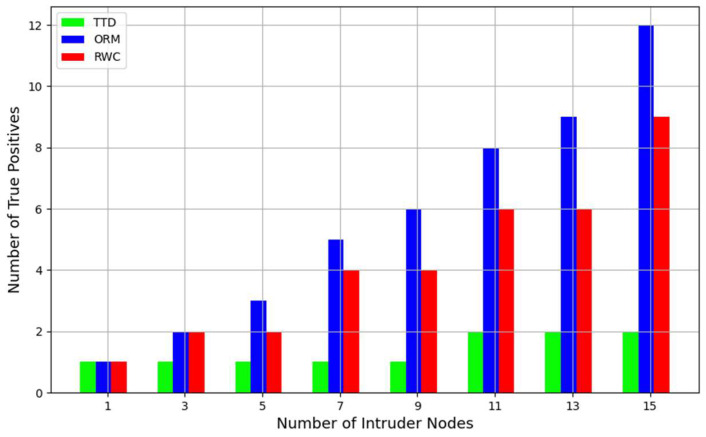
Comparative analysis of true positive recognition in ORM vs. TTD and RWC.

Similar to TTD, we selected 50 nodes for our simulations. TTD considers belief, disbelief, and uncertainty as parameters to distinguish between malicious and benign nodes. In contrast, our proposed ORM is based on honesty, dishonesty, energy, unselfishness, similarity, and neighborhood trust. Figure 4 compares the number of true positives correctly recognized by both approaches as the number of intruder nodes increases up to 15 in the network.

The ORM’s superior performance over the TTD and RWC approaches across various metrics is notably evident in the simulation results. When analyzing true positives (TPs), the ORM demonstrates better accuracy, particularly when confronted with scenarios involving 1, 3, 11, 13, and 15 malicious nodes. These instances highlight the ORM’s efficiency in pinpointing malicious nodes accurately. Conversely, instances with 5, 7, or 9 intruder nodes show comparable performance between both methodologies. However, the ORM shows enhanced accuracy as the number of malicious nodes increases, indicating a consistent performance over the TTD method, as depicted in Figure 4. The ORM’s multifaceted trust calculation involving energy, unselfishness, honesty, dishonesty, and similarity metrics likely enables it to detect a node integrity more precisely than TTD, as reflected by higher accuracy in detecting intruders when the number of malicious nodes varies. Additionally, the results obtained with the RWC method show similar trends to the ORM, with RWC consistently outperforming TTD in terms of true positives across different scenarios, though not surpassing the ORM’s performance.

**Figure 5:**
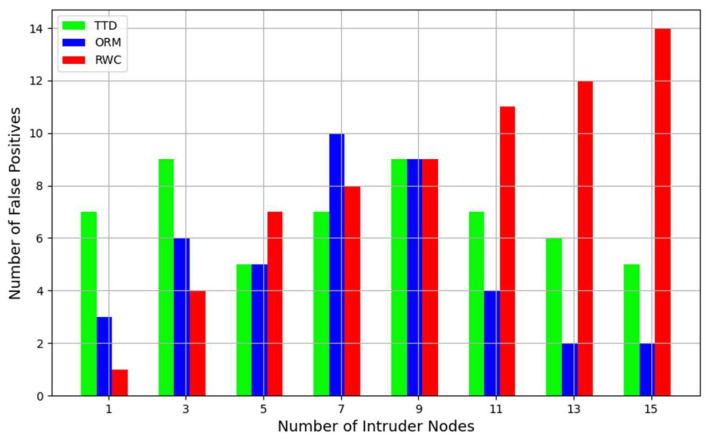
Comparative analysis of false positives in ORM vs. TTD and RWC.

Figure 5 depicts the scenarios where benign nodes are misclassified as malicious. The ORM consistently outperforms TTD across various intrusion scenarios, showcasing its enhanced ability to minimize false positives. This is due to the ORM’s delta-based trust isolation, allowing it to precisely pinpoint and isolate suspicious nodes, thereby reducing the misclassification of benign nodes as malicious entities. Additionally, the ORM’s comprehensive trust evaluation, incorporating multiple metrics, likely contributes to its more refined identification of benign nodes, further minimizing false positives compared to TTD. In comparison to TTD and ORM methods, the RWC method exhibits a notably higher incidence of false positives across varying scenarios with different numbers of intruder nodes. This tendency suggests a potential limitation in RWC’s ability to accurately distinguish between benign and malicious nodes, resulting in a higher rate of misclassifying benign nodes as intruders. While TTD and ORM methods demonstrate more consistent performance, with generally lower false positive rates, RWC’s elevated false positive occurrences underscore its inferiority in intrusion detection within IoT environments. 

While both TTD and ORM methods exhibit good accuracy for true positives and false positives, RWC’s notably higher false positive rate makes it less suitable for intrusion detection tasks in IoT environments. Given the substantial discrepancy in false positive rates and the consistent performance of TTD in true positives and false positives, subsequent analyses will focus solely on comparing the ORM with TTD. This selective comparison allows for a more in-depth examination of the ORM’s efficacy in detecting and mitigating threats compared to the more conventional TTD approach.

**Figure 6:**
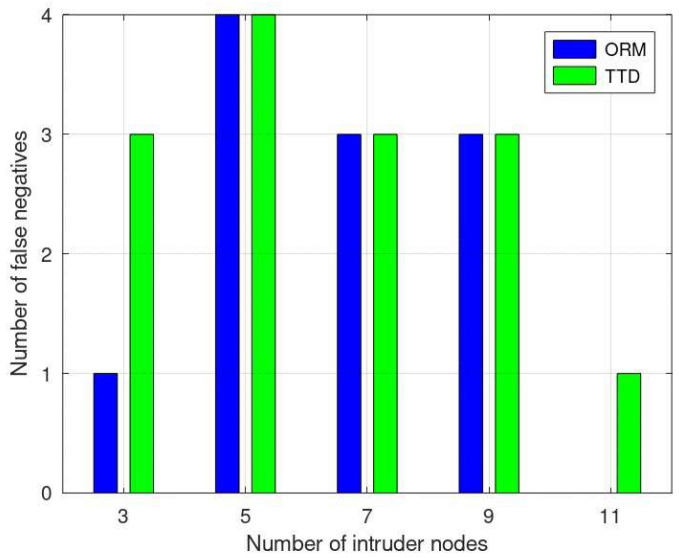
Comparative analysis of false negatives in ORM vs. TTD.

Figure 6 shows the number of false negatives (FNs), where a malicious node is mistakenly categorized as benign. The ORM displays a consistent performance when the number of intruder nodes is 1, 3, 11, 13, and 15. Moreover, both approaches exhibit similar results for 5 and 9 intruder nodes. However, as the number of malicious nodes rises, the ORM’s performance in terms of false negatives notably improves. The ORM’s adaptive nature, especially due to the forgetting curve prioritizing recent behavior, allows for more accurate trust evaluations. This adaptability to changing node behaviors potentially contributes to the lower false negatives and higher accuracy of the ORM in identifying both benign and malicious nodes across diverse scenarios.

**Figure 7:**
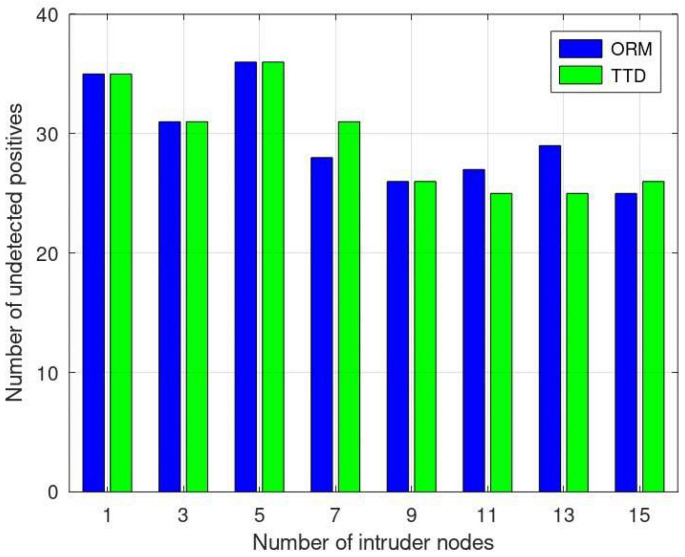
Comparative analysis of undetected positives in ORM vs. TTD.

Undetected positives, as depicted in Figure 7, represent the nodes that could not be classified as benign or malicious due to limited observations. As the number of intruder nodes increases, the performance of the ORM improves. Figure 6 demonstrates that the ORM consistently recognizes fewer undetected positives compared to the TTD, which is especially evident with 7 and 15 intruder nodes. In other cases, both approaches perform similarly in identifying undetected positives.

**Figure 8:**
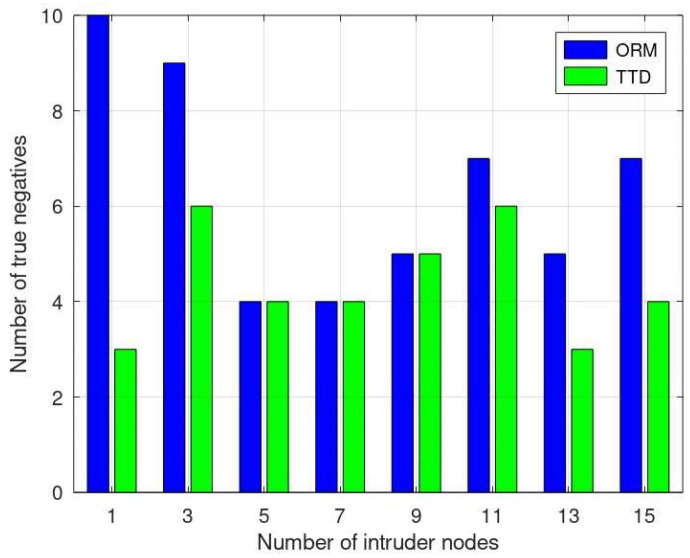
Comparative analysis of true negative evaluation in ORM vs. TTD.

In terms of true negatives (TNs), where benign nodes are accurately identified as benign, both methods perform equally well when the number of malicious nodes is 5, 7, or 9. However, the ORM displays better performance, detecting more benign nodes compared to TTD, specifically for 1, 3, 11, 13, and 15 intruder nodes, as shown in Figure 8. The ORM’s ability to consistently detect benign nodes across varying intrusion instances indicates a potentially enhanced discrimination capability compared to the TTD approach. This capability leads to a reduced number of false negatives, showcasing the ORM’s precision in differentiating benign nodes, even in scenarios with different degrees of intrusion.

**Figure 9:**
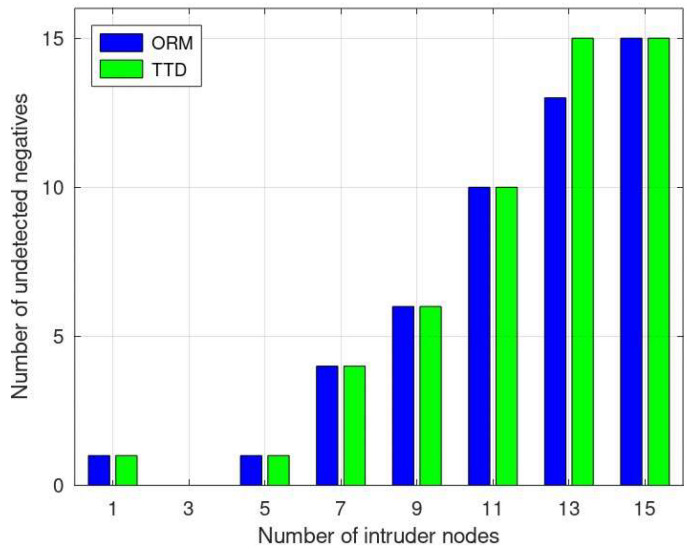
Comparative analysis of undetected negatives in ORM vs. TTD.

In Figure 9, the undetected negatives represent the benign nodes. The identification of such nodes is challenging due to limited observations. However, as reflected in Figure 8, the performance of the ORM is comparable to the other methodologies. The ORM showcases enhanced performance, notably observable in scenarios where the number of malicious nodes reaches 13, as shown in Figure 8. The ORM’s enhanced performance in identifying undetected negatives, especially prominent with 13 malicious nodes, stems from its adaptive trust evaluation. Additionally, the ORM’s comprehensive evaluation, considering multiple trust metrics, likely aids in mitigating misclassifications, contributing to its better identification of benign nodes in challenging scenarios with limited observations.

**Figure 10:**
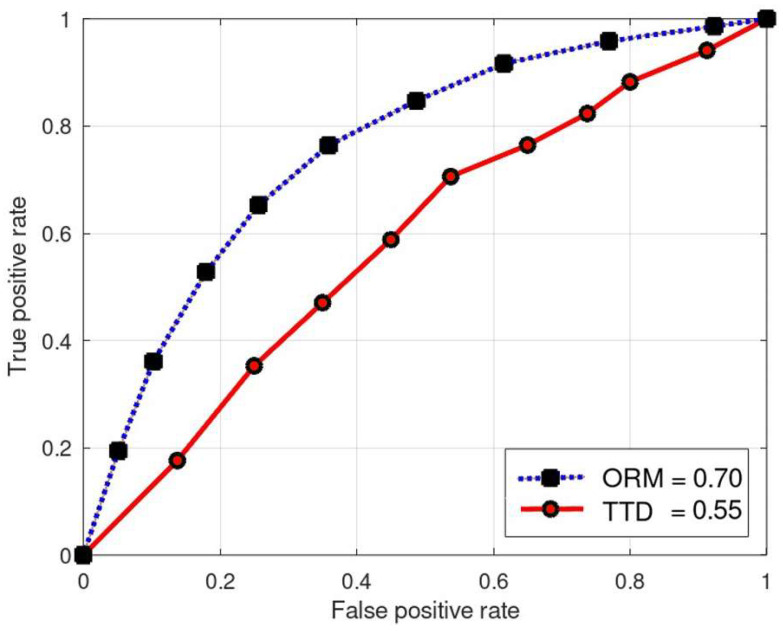
Receiver operator curve—evaluating discrimination power in ORM vs. TTD.

The ROC curve presented in Figure 10 offers a comparative analysis between ORM and TTD methodologies. This curve visualizes the relationship between Sensitivity (True Positive Rate) and False Positive Rate (FPR). Sensitivity measures the accurate classification of class M items, while FPR gauges the misclassification of items not belonging to class M. Additionally, Specificity (True Negative Rate) defines the precision in classifying items not of class M. The area under the curve (AUC) for the ORM is calculated as 0.7, indicating a notably higher probability of detection compared to TTD, which has an AUC of 0.55. This significant discrepancy suggests that TTD struggles to achieve an acceptable detection probability, indicating its limited applicability, especially in IoT systems where RPL implementation is lacking.

## 6. Conclusions

In this paper, we present a delta-based optimized reporting module. Our approach shows significant accuracy in identifying malicious nodes across various intrusion scenarios. Its adaptability feature, resulting in improved trust assessment, and precise isolation methods contribute to reducing false negatives and false positives. Conversely, TTD struggled to achieve satisfactory detection probabilities, making it less practical in real IoT settings. 

The study highlights the important role of adaptable and comprehensive trust evaluation mechanisms, as demonstrated by the ORM, in strengthening network security in dynamic IoT environments. These findings provide valuable insights for enhancing IoT security protocols and lay a robust foundation for future research in this critical domain. However, the proposed model’s reliance on multiple parameters for trust computation raises concerns about resource and communication overhead. Additionally, balancing accuracy against system complexity poses a challenge, potentially impacting manageability and scalability in larger networks. Furthermore, sensitivity to dynamic network conditions adds to its limitations.

In future, we aim to focus on the above-mentioned limitations as well as on optimizing the trust calculation mechanisms to reduce resource and communication overhead in IoT devices while maintaining accuracy. Moreover, we will investigate the integration of machine learning to enhance trust assessment in dynamic network settings. 

## Figures and Tables

**Table 1:** Summary of the existing schemes.

References	Neighbor Overhearing	Leaf Node	False Alarm	Faulty Reporting
[8]	√	√	√	√
[18]	√	√	√	√
[19]	√	√	√	√
[20]	×	×	√	√
[26]	×	×	√	√
[27]	×	√	√	√
[28]	×	×	√	√
[29]	√	√	√	√
[30]	×	×	×	√
[31]	√	√	√	√
[32]	√	√	√	√
[33]	√	√	√	√
[34]	×	√	√	√
[35]	√	√	√	√
[36]	√	√	√	√
[37]	×	√	√	√

## Data Availability

This research did not use any public or private datasets. No new data were generated. All the data were generated in real time as discrete events in a simulator.
